# Biochemical and mutational analyses of *HEXA* in a cohort of Egyptian patients with infantile Tay-Sachs disease. Expansion of the mutation spectrum

**DOI:** 10.1186/s13023-023-02637-1

**Published:** 2023-03-13

**Authors:** Doaa M. A. Ibrahim, Ola S. M. Ali, Hala Nasr, Ekram Fateen, Alice AbdelAleem

**Affiliations:** 1 Department of Medical Molecular Genetics, Human Genetics and Genome Research Institute, National Research Centre, Cairo, Egypt; 2 Department of Biochemistry, Faculty of Pharmacy (Girls), Al-Azhar University, Cairo, Egypt; 3 Department of Biochemical Genetics, Human Genetics and Genome Research Institute, National Research Centre, Cairo, Egypt

**Keywords:** Infantile Tay-Sachs disease, HEXA gene, β-hexosaminidase-A enzyme, HEXA mutation spectrum, Egyptian patients with TSD, Biochemical analysis of HexA-enzyme, Molecular diagnostic, Rare neurodegenerative diseases, Sanger sequencing

## Abstract

**Background:**

Tay-Sachs disease (TSD), an autosomal recessively inherited neurodegenerative lysosomal storage disease, reported worldwide with a high incidence among population of Eastern European and Ashkenazi Jewish descent. Mutations in the alpha subunit of *HEXA* that encodes for the β-hexosaminidase-A lead to deficient enzyme activity and TSD phenotype. This study is the first to highlight the *HEXA* sequence variations spectrum in a cohort of Egyptian patients with infantile TSD.

**Results:**

This study involved 13 Egyptian infant/children patients presented with the infantile form of TSD, ten of the 13 patients were born to consanguineous marriages. β-hexosaminidase-A enzyme activity was markedly reduced in the 13 patients with a mean activity of 3 µmol/L/h ± 1.56. Sanger sequencing of the *HEXA’* coding regions and splicing junctions enabled a detection rate of ~ 62% (8/13) in our patients revealing the molecular defects in eight patients; six homozygous-mutant children (five of them were the product of consanguineous marriages) and two patients showed their mutant alleles in heterozygous genotypes, while no disease-causing mutation was identified in the remaining patients. Regulatory intragenic mutations or del/dup may underlie the molecular defect in those patients showing no relevant pathogenic sequencing variants or in the two patients with a heterozygous genotype of the mutant allele. This research identified three novel, likely pathogenic variants in association with the TSD phenotype; two missense, c.920A > C (E307A) and c.952C > G (H318D) in exon 8, and a single base deletion c.484delG causing a frameshift E162Rfs*37 (p.Glu162ArgfsTer37) in exon 5. Three recurrent disease-causing missense mutations; c.1495C > T (R499C), c.1511G > A(R504H), and c.1510C > T(R504C) in exon 13 were identified in five of the eight patients. None of the variants was detected in 50 healthy Egyptians’ DNA. Five variants, likely benign or of uncertain significance, S3T, I436V, E506E, and T2T, in exons 1, 11,13, & 1 were detected in our study.

**Conclusions:**

For the proper diagnostics, genetic counseling, and primary prevention, our study stresses the important role of Next Generation Sequencing approaches in delineating the molecular defect in TSD-candidate patients that showed negative Sanger sequencing or a heterozygous mutant allele in their genetic testing results. Interestingly, the three recurrent TSD associated mutations were clustered on chromosome 13 and accounted for 38% of the *HEXA* mutations detected in this study. This suggested exon 13 as the first candidate for sequencing screening in Egyptian patients with infantile TSD. Larger studies involving our regional population are recommended, hence unique disease associated pathogenic variations could be identified.

## Introduction

Tay-Sachs disease (TSD) or GM2 gangliosidosis variant B (MIM: 272800) is a rare autosomal recessive neurodegenerative lysosomal storage disorder that is fatal in infancy. TSD is caused by mutations in the alpha-subunit *HEXA* gene (MIM# 606869) of β-hexosaminidase-A enzyme leading to deficiency of the enzyme activity and TSD phenotype. The main pathology of the disease is caused by the progressive accumulation of GM2 ganglioside and associated glycosphingolipids in the lysosomes, mainly that of the neurons promoting progressive neurodegeneration [1]. The clinical presentation is classified according to the age of disease- onset into infantile, juvenile, and adult forms. The most severe form is the classical infantile TSD, which presents initially with motor weakness, increased startle response at 3–5 months of age, and is invariably associated with macular red spots. Neurological signs become increasingly evident with the loss of earlier achieved motor milestones, impaired vision, deafness, feeding problems and seizures. Further deterioration ends in an unresponsive vegetative state and death at 2–4 years of age. This fatal phenotype is common to all patients with deficient β-hexosaminidase-A activity. Later-onset TSD phenotypes are more clinically variable and are usually divided into juvenile and adult forms [2]. Recently, an adeno-associated virus (AAV) gene therapy clinical trial to establish safety dosage and provide infantile TSD patients derived data has shown promising results of a time-limited disease stabilization [3]. TSD disease showed a particularly high incidence in the Ashkenazi Jewish population with a carrier frequency of approximately 1 in 25 compared to 1/250–300 in most other populations. Also, the incidence of patients affected by the disease is estimated to be 1 in 3600 live births in Ashkenazi Jewish versus 1 in 360,000 in other populations worldwide [4, 5]. Population-based carrier screening programs in the United States and Canada reduce the incidence of TSD by more than 90% in these populations [6, 7].

To date > 220 *HEXA* mutations were identified in the Tay-Sachs mutation database [8, 9], *HEXA* mutations may impact the protein’s structure, folding, and/or transport, which ultimately leads to functional impairment [10, 11]. Ethnic-related common gene mutations have been reported in Ashkenazi Jews patients, in 94–98% of them, TSD is caused by one of three common mutations; c.1277_1278 ins TATC in exon 11, 1421 + 1 G > C transition at the splice junction of exon/intron 12 and c.805 G > A (p.G269S) [12]. Non-Ashkenazi TSD patients exhibited rare mutations distributed all over the HEXA gene [13, 14].

In the present study, we were aiming to reveal the *HEXA* mutations’ spectrum and genotypes in Egyptian patients with the infantile form of TSD. Bidirectional direct Sanger sequencing analysis of the entire coding region and splice junctions of the gene was our implemented approach.

## Subjects and methods

### Subjects and samples collection

Twenty-three unrelated individuals were enrolled in this study. Patients were ascertained based on the preliminary clinical findings of early onset progressive neurodegenerative disease associated with seizures, hypotonia, loss of earlier acquired motor skills, cherry red spots, and maybe loss of vision. The confirmatory criterion for inclusion as TSD was the reduced measured HeX-A enzyme activity. Patients were referred by the biochemical genetics department at the National Research Centre (NRC) in Egypt. Enrolled subjects are subdivided into 3 groups: the first group included 13 patients with HeX-A positive assay [enzyme activity was below 5 µmol/L/h], the second group involved 5 patients clinically presented with neurodegenerative manifestations but with normal HeX-A enzyme assay [enzyme activity was in the normal range], and a third group of 5 normal controls. Patients were clinically classified as infantile TSD phenotype, according to the disease’s age of onset. The study was approved by the Institutional Ethics committee of NRC. Written informed consent was obtained from participants or their legal guardians.

### Enzyme assay

Fresh peripheral blood samples were collected on EDTA from each participant, a specific lab code was given for each blood tube. A standard fluorimetric method using the specific sulphated fluorogenic substrate, 4-methylumbelliferyl-N-acetyl-β-D-glucosamine “MUG”, was applied to measure the enzyme activity in serum [15].

### Sanger sequencing of the HEXA gene alpha-subunit

Participants’ genomic DNA extracted from fresh peripheral blood leukocytes using the salting out protocol [16] was quantified and amplified. The 14 exons and splicing junctions of *HEXA* were PCR amplified in 12 fragments using specifically described primers [13]. Each amplicon was purified using shrimp alkaline phosphatase and exonuclease I protocol. Sanger sequencing analysis of every single stranded fragment was performed in forward and reverse directions, using the BigDye^®^ Terminator v3.1 kit (Applied Biosystems). Sequencing reactions were purified and run on the 310 Genetic Analyzer (Applied Biosystems). The occurrence of the sequence variations identified in patients was tested in 50 de-identified healthy Egyptian DNA samples applying the same procedures.

### In-silico assessment of the novel sequence variants

The pathogenicity and frequency of sequence variations identified in this study were assessed using the available public databases including the HGMD [8, 9], HEXAdb, OMIM [18], and online-based prediction tools of VarSome [19] and Ensemble [20].

## Results

### Patients’ characteristics

This study included 18 unrelated Egyptian patients who presented with the provisional clinical diagnosis of a neurodegenerative lysosomal disorder. Patients’ age group falls in the range from birth to ~ 4 years. All patients enrolled in this study were Egyptian, and none of them was of Jewish origin. Parental consanguinity was documented in ten of the 13 confirmed TSD patients (77%). The patient's phenotype was suggestive of neurodegenerative infantile Tay-Sachs disease involving myoclonic seizures, progressive neurological impairment, brisk deep tendon reflex, deafness, and macular degeneration, a Cherry red spot was found in all our Tay Sachs affected patients. Exaggerated Startle reflex to noises was always the first sign noticed by the parents at a median age of onset 7 months ± 3.2, extended into infantile spasms that were usually febrile at the beginning then myoclonic seizures happened without fever. Rapid deterioration of all acquired developmental skills, spasticity of the limbs, and loss of contact/interest in the surroundings associated with loss of vision were the sequence of events. All patients were macrocephalic. The confirmed clinical diagnosis was based on the biochemical result of deficient β-hexosaminidase-A enzyme activity. Affected children died before reaching the age of 24 months except for four patients (3, 6, 7, and 10) that survived to four years old. Five normal control Egyptians were involved in checking for the common neutral single nucleotide polymorphism detected in our patients and reported in other populations. Demographic data of the enrolled subjects are shown in Table 1.

**Table 1:**
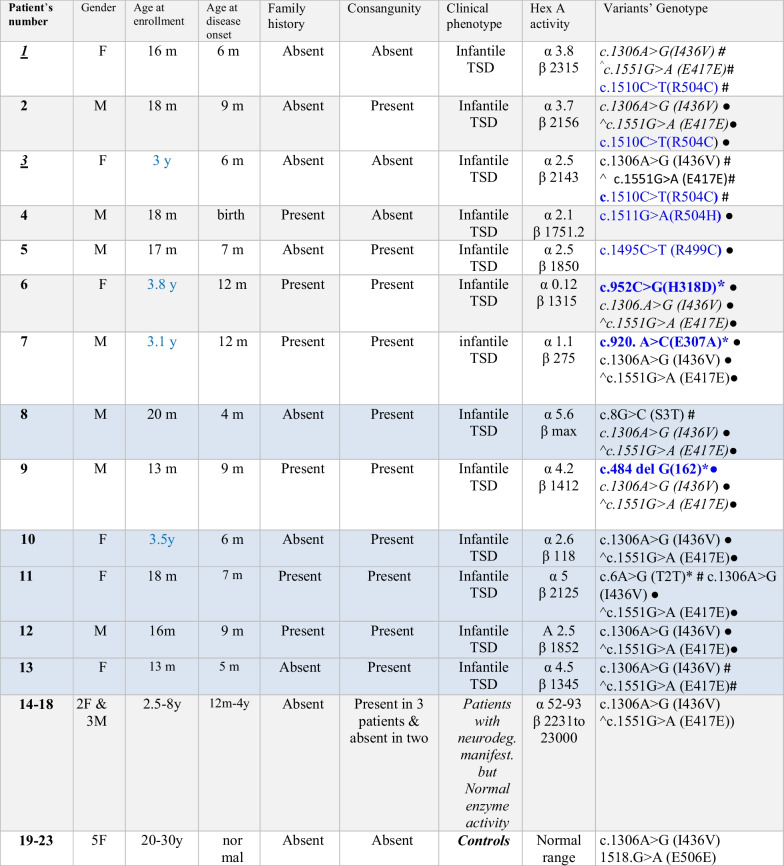
Demographic, biochemical, and sequence variation characteristics in TSD Egyptian patients

*y:* years, *m:* months, *neurodeg:* neurodegenerative, *manifest:* manifestations, *F:* female, *M:* male

^c.1551G > A (E417E) corresponds to c.1518G > A (E506E) in a different transcript

Enzyme activity unit: l µmol/L/h

Normal HEXA- α subunit enzyme activity reference range = 50–200 µmol/L/h

Normal HEXA- β subunit enzyme activity reference range = 100–3500 µmol/L/h

Blue shaded rows present TSD patients with a confirmed deficient Hex-A enzyme activity, but the causative mutations were not identified

*Novel mutations

#Heterozygous variant

●Homozygous variant

### Biochemical findings (Hex-A enzyme activity)

Deficient β-hexosaminidase-A (Hex-A) enzyme activity was confirmed in 13 patients, whereas a normal range of the enzyme activity was shown in five patients, consequently, the diagnosis of TSD was excluded in them.

β-Hexosaminidase-A activity was found markedly reduced in TSD patients compared to the reference values. The enzyme activity ranged between 0.1 and 5.6 µmol/L/h with a mean of 3 µmol/L/h ± 1.56 in the 13 TSD patients (the reference normal range is 50–200 µmol/L/h). A normal range of 52–90 µmol/L/h was detected in the five patients that were clinically ascertained as potential TSD candidates. The values of Hex-A enzyme activity as well as that of the associated lysosomal hydrolase β-hexosaminidase; alpha and beta subunits measured in fresh leukocytes blood samples of participants enrolled in this study are shown in Table1.

### Molecular findings and mutation spectrum in TSD patients

**Fig. 1:**
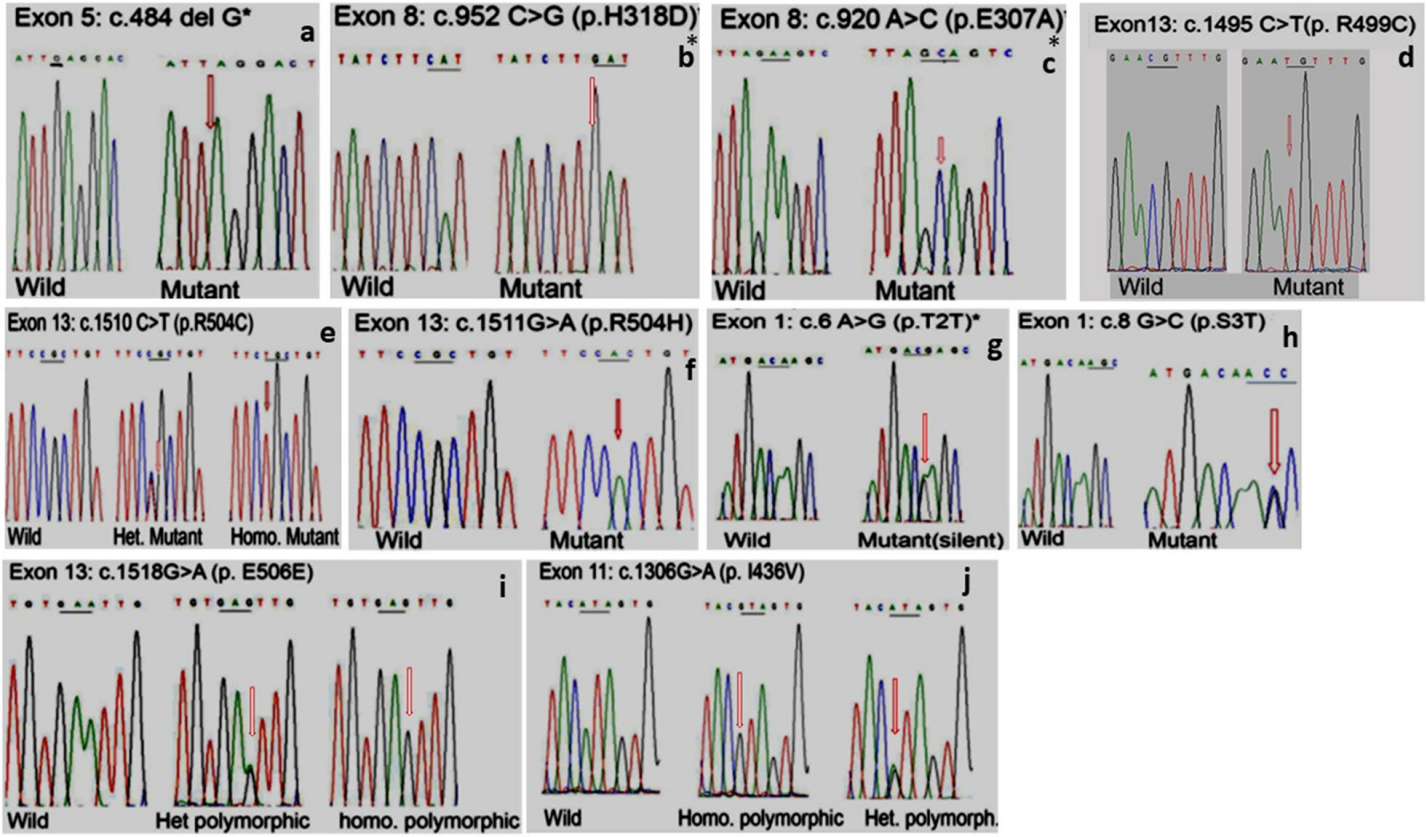
Sanger sequencing chromatographs of *HEXA* gene mutations in Egyptian patients with infantile TSD. The chromatographs showed the mutations identified in nine patients [P1-P9] in exons 5, 8, and 13 as well as the polymorphic variations. **a** (P9): c.484 delG (homozygous); **b** (P6): c.952C > G (P.H318D (homozygous); **c** (P7): c.920A > C (p.E307A) (homozygous); **d** (P5): c.1495C > T (p. R499C) homozygous; **e** (P1,2,3): c.1510C > T (p.R504C) homozygous and heterozygous; **f** (P4): c.1511G > A (p.R504H) (homozygous); **g** (P11): c.6A > G (p.T2T). Silent rare sequence variant. **h** (P8): c.8G > C (p.S3T). Polymorphic variant. **i** (P1-18,C1-5): c.1518.G > A (p.E506E). Silent sequence variant. **j** (P1-18, C1-5): c.1306.G > A (p. I436V). Polymorphic variant. Arrows indicate the locations of the mutations in the amino-acid sequence of α-hexosaminidase A protein. *: Novel variant, c.: c.DNA, p: protein

Sequencing analysis of *HEXA* α-subunit coding and flanking regions detected 15 mutant alleles, out of the anticipated 26 alleles of the 13 TSD cases; six patients were homozygous for the mutant alleles, five of them were the product of consanguineous marriages. While two patients (P1 and P3) were heterozygous for the mutant allele (Fig. 1a–f), and in five TSD patients with deficient enzyme activity, no mutation was identified. Mutations’ details are shown in Tables 1 and 2. The two unrelated patients, P1 and P3, in whom the second allele was not identified are the product of a non-consanguineous parent and carrying the same heterozygous mutation c.1510C > T(R504C) in exon 13, as well as the common polymorphic benign alleles, c.1306A > G (I436V) and c.1551G > A (E417E) in exons 11 and 13, respectively in heterozygous genotypes.

Three Novel, likely pathogenic variants involve a frameshift single nucleotide deletion, c.484delG and two missense variants, c.952C → G (H318D) and c.920A → G (E307A) first detected in the present study and were not previously reported in any of the public databases or in 100 Egyptian chromosomes. Recurrent three variants, R499C (c.1495C → T), R504C (c.1510C → T) and R504H (c.1511G → A) previously reported in association with TSD, were identified also in our patients.

**Table 2:** Distribution of HEXA gene variations in Egyptian patients clinically suggestive of infantile TSD

Subjects	Exon	c.DNA position	Amino acid change	Frequency (%)	Ethnic population	References
3/13 (P1,P2,P3)	Ex13	c.1510C > T g. 72,637,803 C > T	p.R504C (Arg504Cys) CGC˃TGC	23	German, French, Algerian, & Egyptian	Akli et al. (1991) Akli et al. (1993b) Paw et al. (1991) Tanaka et al. (1994) This report
1/13 (P4)	Ex13	c.1511G > A g. 72,637,802 G > A	p.R504H (Arg504His) CGC˃CAC	7.7	Assyria, Lebanes, Americn, & Egyptian	Paw et al. (1990a) This report
1/13 (P5)	Ex13	c.1495C > T g. 72,637,818 C > T	P.R499C (Arg499Cys) CGT˃TGT	7.7	Slavic, Irish, English, Polish, & Egyptian	Akli et al. (1993b); Mules et al. (1992b); Paw et al. (1990); This report
1/13 (P6)	Ex8	c.952C > G g. 72,641,454 C > G	p.H318D (Hist318Asp) CAT˃GAT	7.7	Egyptian	Novel. This report {A different amino acid change (H318R) reported at the same codon}
1/13 (P7)	Ex8	c.920A > C g. 72,641,486 A > C	p.E307A (glu 307ala) GAA˃GCA	7.7%	Egyptian	Novel. This report. A different amino acid change (E307K) reported at the same codon
1/13 (P9)	Ex5	c.484del G g. 72,645,495 delG	p. E162Rfs*37	7.7	Egyptian	Novel. This report
11/13 TSD patients, 5/5 patients with normal Hex-A enzyme & 5/5 controls	Ex11	c.1306A > G g.72638892A > G	p.I436V (Ile 436 Val) ATA˃GTA	91	Egyptian, Black American, & mixed ethnic	Mules et al. (1992b) This report
11/13 TSD patients, 5/5 patients with normal HEXA enzyme & 5/5 controls	Ex13	c.1551A > G g.72637795 G > A {c.1518A > G}	p. E417 = (Glu417 =) GAA˃GAG {P. E506 =}	91	Egyptian, German, & multiethnic	Paw et al. (1991); Akli et al. (1993b); Tanaka et al. (1994); This report
1/13 (P8)	Ex1	c.8G > C g.72668306G > C	p.S3T (Ser3Thr) AGC˃ACC	7.7	Egyptian, Ashenazi J. &mixed ethnic	Cecchi et al. (2019); This report
1/13 (P11)	Ex 1	c.6A > G g. 72,668,308	P.T2T ACA > ACG		Egyptian, No data for the ClinVar submission	This report; a ClinVar submission

### In-silico pathogenicity prediction of the novel variants

- The frameshift, c.484delG, E162Rfs*37 predicted of a high impact on the encoded protein and resulting in nonsense mediated decay. The c.484delG has not been reported in gnomAD or other public databases.
- The missense H318D variant: ACMG classification is likely pathogenic based on:
  - The position is very highly conserved, score 9.7 by phyloP100 vertebrates, not reported in GnomAD exomes or genomes, 11 pathogenic prediction tools predict this variant as damaging/deleterious/or disease causing, and almost always missense mutations that is not variant of uncertain significance (VUS) in *HEXA* is disease causing.
- The missense E307A variant: ACMG classification is likely pathogenic conservation score = 7.9 by PhyloP100, reported as damaging/deleterious/or disease-causing in 11 pathogenicity prediction tools, and not found in GnomAD Exomes or Genomes.

### ***Polymorphic variants*** (Fig. 1g–j)

Four *HEXA* polymorphic variants were identified in the present study. The c.1551A > G(E517E) and c.1306A > G (I436V) were commonly described. The c.1551A > G (p.Glu517 =) in exon 13, (Transcript NM_001318825.2) has a GnomAD Exomes frequency of  0.97 and  classified as benign. This variant corresponds to c.1518A > G, (p.Glu506 =) in transcript NM_000520.6.

The c.1306A > G, I436V (p. Ile436Val) in exon 11, classified as VUS in a single database submission (Transcript NM_001318825.2) and as benign in multiple submissions (NM_000520.6). GnomAD Exomes frequency = 0.971.

The c.6A > G (T2T) silent substitution in exon 1, was newly reported in this study in association with TSD. There was a single clinical testing submission of T2T on CliniVar, but no assertion criteria were provided, and the TSD-affected status was unknown. The frequency of this variant is very rare; in gnomAD exomes = 0.0000319, in gnomAD genomes ƒ = 0.000004.

The fourth variant, c.8G > C (*S3T*) classified as likely benign; the position is not conserved (phyloP100 = 0.297), while predicted as damaging in only two prediction tools. However, it is of the rare alleles, its allele frequency in gnomAD exomes ƒ = 0.00046, and in gnomAD genomes ƒ = 0.000255. The S3T has never been reported as homozygous in databases, only as heterozygous with a normal enzymatic assay association.

## Discussion

TSD patients ascertained in the present study displayed developmental delay, hypotonia, loss of motor milestones, seizures, deafness, and the presence of a cherry red spot in fundus examination. The markedly reduced Hex-A enzyme activity (mean 3 µmol/L/h ± 1.56) has confirmed the diagnosis of TSD.

This study is the first to uncover the *HEXA* mutations’ spectrum and correlate the β-hexosaminidase-A enzyme deficiency to the underlying *HEXA* molecular defects in a homogeneous population of Egyptian patients with the infantile form of TSD. The sequencing technology applied here, Sanger sequencing of the coding and splice junction regions of the gene enabled the identification of the molecular defect in ~ 62% of patients enrolled, in two of them a single mutant allele was detected. While in ~ 38%, the molecular pathology remained undiagnosed.

The characteristics of patients 1 and 3 in whom the same recurrent mutation c.1510C > T(R504C), as well as the two polymorphic benign alleles, c.1306A > G (I436V) and c.1551G > A (E417E) were all in heterozygous genotypes seem interesting. It may suggest a potential haplotype of the TSD disease-causing mutation (in exon13) and two common variants in exons 11&13. This heterozygous haplotype may result from an associated chromosomal rearrangement events. It will be interesting to investigate potential cytogenetic events in these two patients, the outcome may support a different mechanism of the molecular pathology that underlies the TSD clinical and biochemical phenotype.

The molecular defect in P8, born to a consanguineous family and had a confirmed Hex-A enzyme deficiency, remains unresolved. Three variants were detected in this patient; the heterozygous missense variant c.8G > C (S3T) that was classified as likely benign, however of a rare frequency (ƒ = 0.00046 in gnomAD exomes), and the two frequently occurring variants in exons 11&13, both were in a homozygous genotype. The same situation of the unrevealed genetic defect was encountered in four patients (P10-13) in whom only the polymorphic variants in exons 11&13 and a silent amino acid change in P11 were detected in their blood derived DNA.

*HEXA* is the only gene, up to date, described in association with TSD, this highlights the importance of NGS technology, WGS, for the detection of the full spectrum of the associated disease-causing variants involving intragenic, deep intronic or copy number variations in TSD patients that proved mutations’ negative in Sanger sequencing. NGS will also facilitate the differential diagnosis promoted by the notable clinical overlap seen in the clinic among infants and children presented with a phenotype suggestive of neurometabolic or neurodegenerative storage diseases.

To date, more than 220 HEXA mutations were reported causing different forms of TSD. Documented mutations involved single-base substitutions, small deletions, small duplications/insertions, partial gene deletions, splicing alterations, and complex gene rearrangements [8, 9, 17, 18]. Mutations detected in the infantile form of TSD are generally located in residues proximal to or involve a functionally important active site or dimer interface [21, 22].

Among the six disease-causing mutations identified in this study, three were originally detected in our patients [E307A, H318D, and E162Rfs*37] and another three have been previously reported [R504C, R504H, and R499C] in different ethnic populations pointing out their diversity. [23–28]. Mutations in exon 13 [R504C, R504H, and R499C] constitute 38.4% of the mutations detected in the present cohort of Egyptian patients (5/13) with infantile TSD, whereas missense mutations in exon 8 [H318D and E307A] present 15.4%. The nucleotide deletion in exon 5 [c.484delG] constitutes 7.7%. These sequencing findings lead us to suggest *HEXA*-exon13 as the first candidate for molecular screening in Egyptian patients with infantile TSD.

The novel missense c.952C > G mutation (H318D) in exon 8, homozygous in P6, falls within residues 192–402 of the enzyme’s alpha subunit. These residues were found to be involved in the hydrolysis of the GM2-ganglioside [29]. Histidine residue is the most common amino acid involved in protein active sites [30]. His318 residue is located within four residues forming D322, a potential active site. A mutation at this site leads to abnormal protein retention and degradation within the endoplasmic reticulum [31]. A missense mutation involving  the same codon H318 was reported in non-Jewish TSD carrier from Germany; however, the substitution was for Arginine H318R [32]. The 2nd novel mutation in exon 8, glutamate substitution for alanine (E307A) at codon 920 was homozygous in P7. Glutamate is a negatively charged, polar amino acid that is frequently involved in protein active sites. Alanine is a non-polar small-size residue with a very short non-reactive side chain. Substitution of a small side chain for a large one can be damaging [30]. Studies that investigated the *HEXA* mutations affecting the α-subunit candidate active site residues, reported the occurrence of severe impairments of the enzyme catalytic activity, which was found to interfere with α-subunit maturation, when introducing substitution of E307 to another residue [33]. A missense mutation at the same 307codon exchanging amino acid glutamate into lysine (E307K) was  previously reported in association with TSD, further supporting the pathogenicity of E307A [34]. The homozygous base pair deletion, c.484 delG, the first base in codon 162 (gAG) in exon 5 of P9, originally described here, leads to a frameshift and premature stop codon and apparently early mediated decay of the encoded protein.

The S3T missense heterozygous variant detected in P8 is positioned as part of the residues of α-subunit that seems to have a role in the enzyme activity against the specific sulfated substrate 4MUGS. S3T has been detected in a screening study for TSD carriers [35]. Earlier studies demonstrated these two domain-residues of α1-191 and α 403-529 to account for enzyme activity against 4MUGS [29]. However, other investigators pointed residues 1–131 as not being involved in binding the negatively charged substrates, instead, they suggested residues 132–283 as the α-subunit negatively charged substrates-binding site [36].

The recurrent R499C mutation was identified as homozygous in P5, the substitution is likely to interrupt the specific disulfide bond and disrupt the three-dimensional structure of the enzyme [37]. The other two mutations R504H and R504C affect the ability of the alpha subunit to dimerize.

R504C was detected as heterozygous in P1 & P3 and homozygous in P2. R504H was homozygous in P4. R504 of the α-subunit directly binds to D494 of the β-subunit in the α-β heterodimer model. Substitution of R504 to C or H causes a disruption of that binding leading to conformational changes in the dimer interface [38]. Frequent recurrence of Arg to His/Cys substitution at positions 499 and 504 is probably a consequence of a mutagenic hot spot of the CpG dinucleotides [29, 33].

The polymorphic variants I436V and E506E were detected in 91% of our patients and control subjects. I436V was frequently reported in normal American black population [14, 39], and also found to occur at high frequency among Ethiopian Jews (heterozygotes frequency: 26.1%), while at a lower frequency in Jewish from eight populations, Iraq, Syria, Tunis, Morocco, Libya, Yemen, Persia and Eastern Europe (1.5–7.0%) [40].

In this study, 77% of patients were the product of consanguineous marriages. The cConsanguinity rate in Egypt reported to be above 30% throughout the last 40 years [41]. In an earlier biochemical study, 58% of Egyptian patients with GM2 gangliosidosis were diagnosed as Tay Sachs disease by enzymatic assay. Parental consanguinity was present in 73.3% of these cases. [42]. Consanguinity is a principal factor in increasing the incidence of rare autosomal recessive diseases among the Arab population. The incidence of Tay-Sachs disease has been markedly reduced by more than 90% since the introduction of carrier screening and counseling in Jewish population in comparison to the non-Jewish who is now at 3–4-fold higher frequency [6, 7].

## Conclusions

The *HEXA* mutations R504C, R504H, and R499C are confirmed as being not specific to the Jewish population but of a diverse background. The novel mutations E307A, H318D, and E162Rfs*37 may be specific to Egyptian or Arab TSD patients, hence additional studies in Arab and Middle East populations are highly recommended for the delineation of unique ethnic-related disease-causing variants. The findings of this study suggest Sanger sequencing of *HEXA*-exon 13, as the first screening mutational target in suspected TSD Egyptian patients. Mutation identification in a proband will be the basic measure in the prevention of TSD recurrence in Egyptian families, the reason the NGS should be considered in patients with negative Sanger sequencing results.

## Data Availability

Sequencing and biochemical data generated in this study are available upon a reasonable request made to the corresponding authors.
